# Supplementary material for: Long-Term Outcomes of 5-Fluorouracil-Related Early-Onset Toxicities: A Retrospective Cohort Study
Source: Cancers (Basel). 2024 Dec 3;16(23):4050. doi: 10.3390/cancers16234050 (PMC11639909; doi:10.3390/cancers16234050)
Supplement: Supplementary file 1 [file cancers-16-04050-s001.zip › cancers-3292339-supplementary.pdf]

Table S1. Toxicities identified based on ICD-10 codes

ICD-10 code	ICD-10 description	Mapped toxicity
K52.1	Toxic gastroenteritis and colitis	Diarrhea
K52.9	Noninfective gastroenteritis and colitis, unspecified	Diarrhea
K58.9	Irritable bowel syndrome without diarrhea	Diarrhea
R19.7	Diarrhea, unspecified	Diarrhea
E86.0	Dehydration	Diarrhea
R11	Nausea and vomiting	Vomits
K12.3	Oral mucositis (ulcerative)	Mucositis
K13.7	Other lesions of oral mucosa	Mucositis
T45.1X1	Poisoning by antineoplastic and immunosuppressive drugs, accidental (unintentional)	Drug poisoning
I20	Angina pectoris	Cardiovascular
I21	Acute myocardial infarction	Cardiovascular
I22	Subsequent myocardial infarction	Cardiovascular
I23	Certain current complications following acute myocardial infarction	Cardiovascular
I24	Other acute ischemic heart diseases	Cardiovascular
I60	Nontraumatic subarachnoid hemorrhage	Neurological
I61	Nontraumatic intracerebral hemorrhage	Neurological
I62	Other nontraumatic intracranial hemorrhage	Neurological
I63	Cerebral infarction	Neurological
I64	Stroke, not specified as hemorrhage or infarction	Neurological
I65	Occlusion and stenosis of precerebral arteries, not resulting in cerebral infarction	Neurological
I66	Occlusion and stenosis of cerebral arteries, not resulting in cerebral infarction	Neurological
I67	Other cerebrovascular diseases	Neurological
I68	Other cerebrovascular disorders in diseases classified elsewhere	Neurological
I69	Sequelae of cerebrovascular disease	Neurological
G40	Epilepsy	Neurological
G41	Status epilepticus	Neurological
G45	Transient cerebral ischemic attacks and related syndromes	Neurological
G46	Vascular syndromes of brain in cerebrovascular diseases	Neurological

Table S2. Toxicities identified based on laboratory values

Laboratory	Threshold	Mapped toxicity
White blood cell count	Below 1000 cells per mm^3	Neutropenia
Platelet count	Below 100,000 cells per mm^3	Thrombocytopenia
Chemistry panel	Below each laboratory lower limit of normal	Hypokalemia
Troponin test	Any request of a troponin test	Cardiovascular

Table S3. Toxicities identified based on procedures performed after initiation of chemotherapy

Procedure	Mapped toxicity
Cardiac catheterization, coronary angiography, heart angioplasty, cardiac stent placement, or percutaneous coronary intervention	Cardiovascular
Computed tomography (CT) or magnetic resonance imaging (MRI) of brain	Neurological

Table S4. ICD-10 cancer diagnosis mapping

ICD-10 code	ICD-10 description	Mapped cancer type
C15	Malignant neoplasm of esophagus	Esophagus
C16	Malignant neoplasm of stomach	Stomach
C17	Malignant neoplasm of small intestine	Small intestine
C18	Malignant neoplasm of colon	Colorectal
C19	Malignant neoplasm of rectosigmoid junction	Colorectal
C20	Malignant neoplasm of rectum	Colorectal
C22	Malignant neoplasm of liver and intrahepatic bile ducts	Biliary tract
C23	Malignant neoplasm of gallbladder	Biliary tract
C24	Malignant neoplasm of other and unspecified parts of biliary tract	Biliary tract
C25	Malignant neoplasm of pancreas	Pancreas
C21	Malignant neoplasm of anus and anal canal	Other sites
C26	Malignant neoplasm of other and ill-defined digestive organs	Other sites
Other	Various other malignant neoplasms	Other neoplasms

Table S5. ICD-10 baseline characteristics mapping

ICD-10 code	ICD-10 description	Mapped comorbidity
I10	Essential (primary) hypertension	Arterial hypertension
I11	Hypertensive heart disease	Arterial hypertension
I12	Hypertensive renal disease	Arterial hypertension
I13	Hypertensive heart and renal disease	Arterial hypertension
I15	Secondary hypertension	Arterial hypertension
I20	Angina pectoris	Ischemic heart disease
I21	Acute myocardial infarction	Ischemic heart disease
I22	Subsequent myocardial infarction	Ischemic heart disease
I23	Certain current complications following acute myocardial infarction	Ischemic heart disease
I24	Other acute ischemic heart diseases	Ischemic heart disease
I25	Chronic ischemic heart disease	Ischemic heart disease
I50	Heart failure	Heart failure
I60	Nontraumatic subarachnoid hemorrhage	Cerebrovascular disease
I61	Nontraumatic intracerebral hemorrhage	Cerebrovascular disease
I62	Other nontraumatic intracranial hemorrhage	Cerebrovascular disease
I63	Cerebral infarction	Cerebrovascular disease
I64	Stroke, not specified as hemorrhage or infarction	Cerebrovascular disease
I65	Occlusion and stenosis of precerebral arteries, not resulting in cerebral infarction	Cerebrovascular disease
I66	Occlusion and stenosis of cerebral arteries, not resulting in cerebral infarction	Cerebrovascular disease
I67	Other cerebrovascular diseases	Cerebrovascular disease
I68	Other cerebrovascular disorders in diseases classified elsewhere	Cerebrovascular disease
I69	Sequelae of cerebrovascular disease	Cerebrovascular disease
G45	Transient cerebral ischemic attacks and related syndromes	Cerebrovascular disease
G46	Vascular syndromes of brain in cerebrovascular diseases	Cerebrovascular disease
G40	Epilepsy	Epilepsy
G41	Status epilepticus	Epilepsy
N18	Chronic kidney disease (CKD)	Chronic Kidney disease

Figure S1. States with oncology centers included in the Guardian Research Network

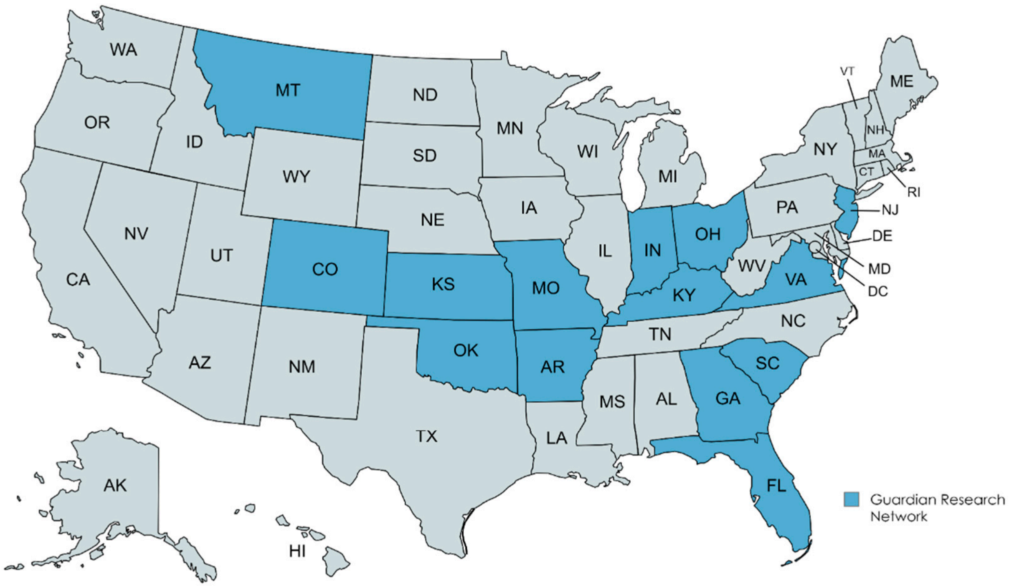

Figure S2. Causal acyclic directed graph

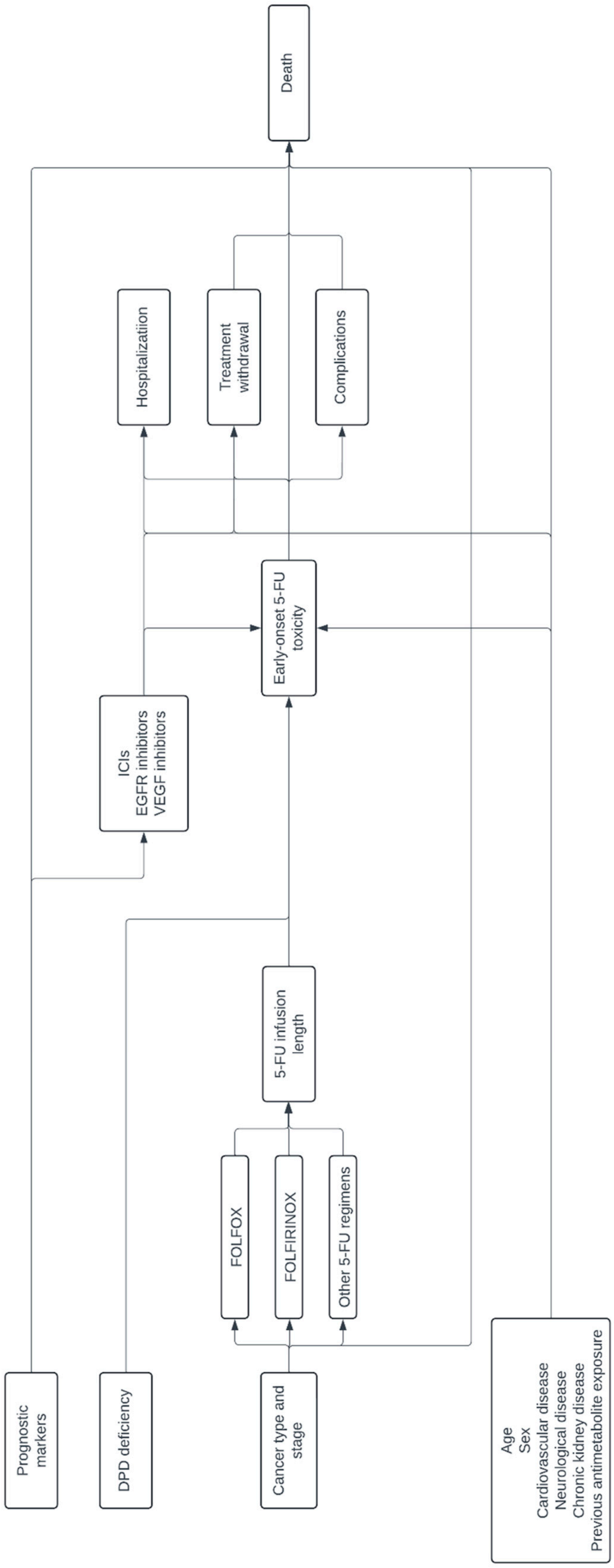

Figure S3. Overall survival by early-onset toxicity occurrence in patients treated with the FOLFOX regimen

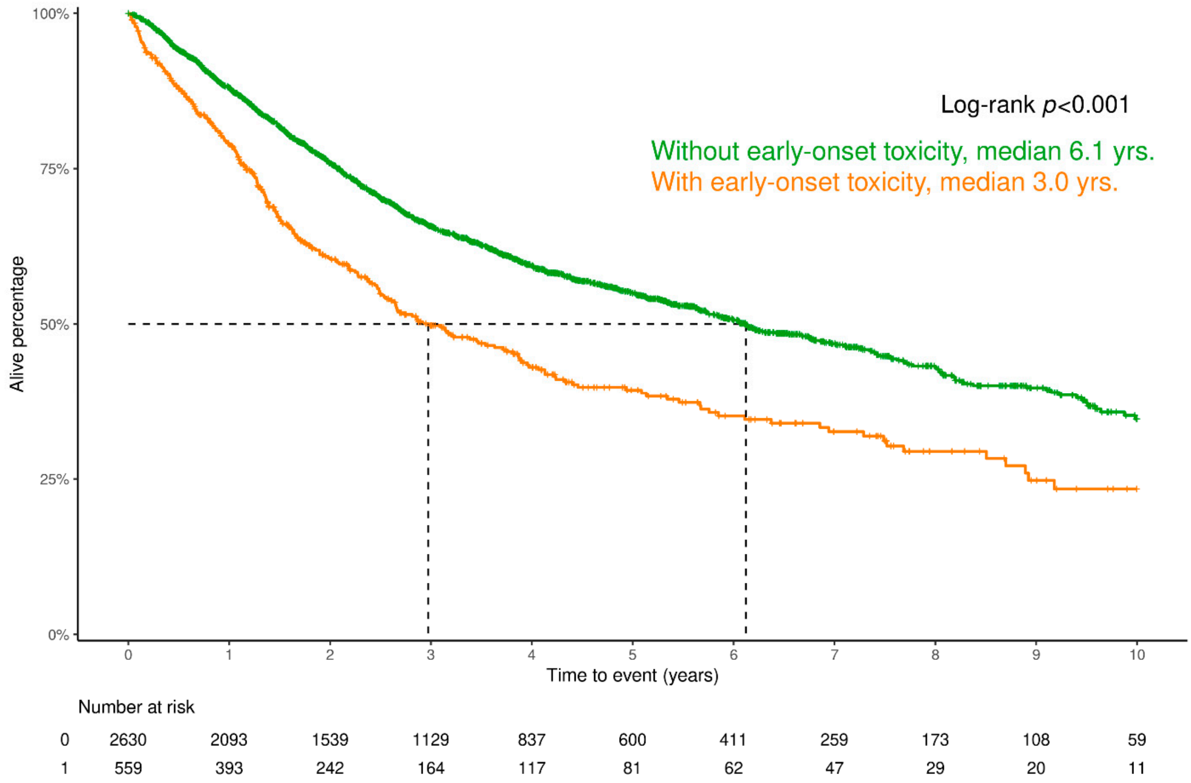

Figure S4. Overall survival by early-onset toxicity occurrence in patients treated with the FOLFIRINOX regimen

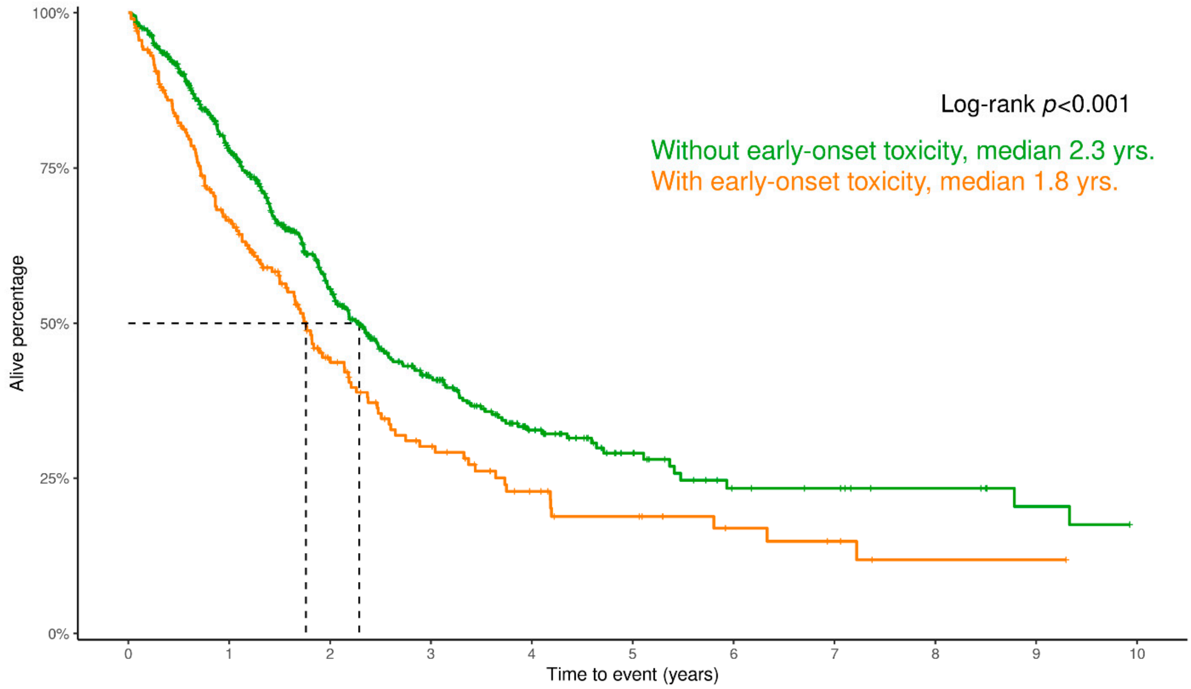

Number at risk

0	595	377	204	106	56	31	17	15	11	7	5
1	204	118	57	33	19	13	8	6	3	3	2
